# Enhancement of NK Cell Antitumor Effector Functions Using a Bispecific Single Domain Antibody Targeting CD16 and the Epidermal Growth Factor Receptor

**DOI:** 10.3390/cancers13215446

**Published:** 2021-10-29

**Authors:** Elisa C. Toffoli, Abdolkarim Sheikhi, Roeland Lameris, Lisa A. King, Amanda van Vliet, Bruce Walcheck, Henk M. W. Verheul, Jan Spanholtz, Jurriaan Tuynman, Tanja D. de Gruijl, Hans J. van der Vliet

**Affiliations:** 1 Amsterdam Infection and Immunity Institute, Cancer Center Amsterdam, Department of Medical Oncology, Amsterdam UMC, Vrije Universiteit Amsterdam, 1081 HV Amsterdam, The Netherlands; e.toffoli@amsterdamumc.nl (E.C.T.); sheikhi@queensu.ca (A.S.); r.lameris@amsterdamumc.nl (R.L.); l.king@amsterdamumc.nl (L.A.K.); td.degruijl@amsterdamumc.nl (T.D.d.G.); 2 School of Medicine, Dezful University of Medical Sciences, Department of Immunology, Dezful 64616-43993, Iran; 3 Glycostem Therapeutics, 5349 AB Oss, The Netherlands; amanda@glycostem.com (A.v.V.); jan@glycostem.com (J.S.); 4 Department of Veterinary and Biomedical Sciences, University of Minnesota, Saint Paul, MN 55108, USA; walch003@umn.edu; 5 Radboud Institute for Health Sciences, Department of Medical Oncology, Radboud University Medical Center, 6525 GA Nijmegen, The Netherlands; Henk.Verheul@radboudumc.nl; 6 Amsterdam UMC, Vrije Universiteit Amsterdam, Department of Surgery, 1081 HV Amsterdam, The Netherlands; j.tuynman@amsterdamumc.nl; 7 Lava Therapeutics, 3584 CM Utrecht, The Netherlands

**Keywords:** NK cells, single domain antibodies, bispecific VHH, EGFR, CD16

## Abstract

**Simple Summary:**

Strategies to enhance the preferential accumulation and activation of Natural Killer (NK) cells in the tumor microenvironment can be expected to increase the efficacy of NK cell-based cancer immunotherapy. In this study, we report that a bispecific single domain antibody (VHH) that targets CD16 (FcRγIII) on NK cells and the epidermal growth factor receptor (EGFR) on tumor cells can be used to target and enhance cytolysis of cancer cells. The bispecific VHH enhanced NK cell activation and cytotoxicity in an EGFR- and CD16-dependent and KRAS-independent manner. Moreover, the bispecific VHH induced stronger activity of cancer patient-derived NK cells and resulted in tumor control in a co-culture of metastatic colorectal cancer cells and either autologous peripheral blood mononuclear cells or allogeneic CD16^+^ NK cells. We believe that this novel approach could represent a valid therapeutic strategy either alone or in combination with other NK cell-based therapies.

**Abstract:**

The ability to kill tumor cells while maintaining an acceptable safety profile makes Natural Killer (NK) cells promising assets for cancer therapy. Strategies to enhance the preferential accumulation and activation of NK cells in the tumor microenvironment can be expected to increase the efficacy of NK cell-based therapies. In this study, we show binding of a novel bispecific single domain antibody (VHH) to both CD16 (FcRγIII) on NK cells and the epidermal growth factor receptor (EGFR) on tumor cells of epithelial origin. The bispecific VHH triggered CD16- and EGFR-dependent activation of NK cells and subsequent lysis of tumor cells, regardless of the KRAS mutational status of the tumor. Enhancement of NK cell activation by the bispecific VHH was also observed when NK cells of colorectal cancer (CRC) patients were co-cultured with EGFR expressing tumor cells. Finally, higher levels of cytotoxicity were found against patient-derived metastatic CRC cells in the presence of the bispecific VHH and autologous peripheral blood mononuclear cells or allogeneic CD16 expressing NK cells. The anticancer activity of CD16-EGFR bispecific VHHs reported here merits further exploration to assess its potential therapeutic activity either alone or in combination with adoptive NK cell-based therapeutic approaches.

## 1. Introduction

Natural Killer (NK) cells are large granular lymphocytes with the innate ability to kill cells that present a threat to the host, such as cancer and virus-infected cells [1,2]. Human NK cells represent ~15% of all peripheral blood lymphocytes and are characterized by the expression of CD56 and the lack of the CD3-T cell receptor complex [3]. Two main NK cell subsets with distinct functions can be defined based on the expression of CD56 and the Fcγ receptor (FcγR)IIIA (CD16a): the CD56^dim^CD16^+^ subset which represents ~90% of human NK cells and is characterized by a more potent cytotoxic activity and the CD56^bright^CD16a^−/dim^ subset described to have lower cytotoxic capacity but to be a stronger producer of immunoregulatory cytokines [3]. NK cell activity is regulated by a balance of inhibitory and activating receptors present on their surface [4]. The former comprises the killer cell immunoglobulin-like receptors (KIR)2D and the natural killer group 2 member (NKG2)A which recognize human leukocyte antigens (HLA), that can be downregulated by tumor and virus-infected cells to escape T cell recognition, and non-HLA-recognizing receptors, such as the Lectin-like Transcript-1 (LLT-1), which binds to NKRP1A. The latter include NKG2D and natural cytotoxicity receptors (NCRs), such as NKp30/44/46, and the DNAX Accessory Molecule-1 (DNAM-1), which bind to specific ligands, such as MHC class I polypeptide–related sequence A/B (MICA/B), UL16 binding protein 1–6 (ULBP1-6), heparan sulfate proteoglycans (HSPG), poliovirus receptor (PVR), and nectin that are overexpressed by infected or malignant cells [1,4]. When the equilibrium of the NK cell receptors is skewed towards activation, due to increased expression of activating ligands or lack of inhibitory signals, NK cells are triggered to release cytotoxic granules and pro-inflammatory cytokines, such as Interferon (IFN)γ and Tumor Necrosis Factor (TNF) [4,5]. Moreover, through the expression of CD16a, NK cell activation can also be triggered by the Fc tail of IgG_1_ antibodies leading to antibody-dependent cell-mediated cytotoxicity (ADCC) [6]. In the last years, the transfer of allogeneic NK cells as a therapeutic cancer strategy has caught the attention of the scientific community due to the anticancer activity of NK cells and the absence of graft versus host disease (GvHD) [7,8,9]. However, preliminary efficacy results are limited, suggesting the need for combining the transfer of NK cells with NK function-augmenting products to achieve a maximum antitumor effect [6,10]. Single domain antibodies (VHHs) are small (~15 kDa) antigen binding fragments derived from heavy chain-only antibodies naturally occurring in Camelidae (e.g., llama) [11]. VHHs have several advantageous properties, including high physicochemical stability, high solubility, a small size, that allows the recognition of hidden antigenic sites and rapid tissue penetration, and low inherent immunogenicity [12,13]. Finally, the production of VHHs has relatively low costs and is time efficient [12,13]. In this study, we describe the functional characterization of a novel bispecific VHH created by the fusion of two previously generated monospecific VHHs: C21 directed against the IgG Fc receptor CD16 and previously shown to trigger NK cell-related IFNγ production [14], and 7D12 which is directed against the epidermal growth factor receptor (EGFR) and interferes with ligand binding, the sterical EGFR transition into the activating conformation, and subsequent signaling [15,16]. CD16 exists in two isoforms: CD16a and CD16b. CD16a can be expressed by NK cells and various T cell subsets, monocytes, and macrophages while CD16b is expressed by neutrophils. CD16 binds the Fc domain of antibodies (Abs) to mediate ADCC, phagocytosis, endocytosis, and/or cytokine release [17,18]. The C21 VHH can recognize and specifically bind both CD16a and CD16b [14]. EGFR is a transmembrane tyrosine kinase receptor normally expressed by epithelial cells in which it is involved in cell proliferation, survival, and motility. Aberrant activation of this receptor, shown in various epithelial cancers, can be caused by receptor overexpression, gene amplification, and activating mutations [19]. Currently, four monoclonal antibodies (mAb) targeting the extracellular domain of EGFR are approved for clinical use against various cancers of epithelial origin (e.g., colorectal cancer (CRC) and head and neck cancer) [20]. However, their clinical efficacy is limited by various factors, such as the presence of activating mutations of the EGFR downstream signaling pathway (e.g., RAS mutations), which result in the constitutive activation of the pathway regardless of ligand binding [21,22,23], the tumor microenvironment (TME), which can restrict antibody penetrance and NK cell migration into the tumor, and/or the presence of certain modulatory non-coding RNAs that hamper the negative modulation of the Wnt signaling pathway leading to EGFR-mAb resistance [20]. In this study, we report the generation of a novel C21/7D12 bispecific VHH and use in vitro and ex vivo studies using patient PBMC and colorectal cancer samples to demonstrate that this bispecific VHH can induce a strong NK cell effector response of both autologous and allogeneic NK cells against EGFR-expressing tumors independent of tumor RAS mutation status. The anticancer activity of this CD16-EGFR bispecific VHH merits further exploration to assess its potential therapeutic activity either alone or in combination with adoptive NK cell-based therapeutic approaches.

## 2. Material and Methods

### 2.1. Generation of Two Bispecific Anti-EGFR-Anti-CD16 VHH Constructs

To generate anti-CD16-anti-EGFR bispecific VHHs, genes encoding the anti-CD16 VHH C21 [14] and the anti-EGFR VHH 7D12 [16] were linked by a Gly4Ser-linker and synthesized (GeneArt, Thermo Fisher Scientific, Waltham, MA, USA) in two orientations (C-7 and 7-C where the N-terminus of the linker was attached to C21 and 7D12, respectively) and cloned into the pHLsec vector with the addition of C terminal hexaHis tag (pHLsec-C21-G4S-7D12-hexaHis and pHLsec-7D12-G4S-C21-hexaHis). Bispecific VHH protein was expressed in HEK293T cells and purified by cobalt affinity, as described previously [24]. The bispecific VHHs were kept in Phosphate-buffered saline (PBS, Fresenius Kabi, Bad Homburg vor der Höh, Germany) at 4 °C or −20 °C for long term storage. After production, batch quality controls were performed by testing the ability of the newly produced bispecific VHHs to enhance NK cell cytotoxicity in comparison to the previously produced batch.

### 2.2. Cell Lines

A431 (epidermoid carcinoma), HCT116 (CRC), and Colo829 (melanoma) cell lines were obtained from the American Type Culture Collection (ATCC, Manassas, VA, USA). A431 and HCT116 were cultured in Dulbecco’s modified medium (DMEM, Gibco, Thermo Fisher Scientific, Waltham, MA, USA) and Colo829 was cultured in Roswell Park Memorial Institute medium (RPMI, Gibco, Thermo Fisher Scientific, Waltham, MA, USA). Culture media were supplemented with 10% Fetal Calf Serum (FCS, Integro, Zaandam, The Netherlands), 100 U/mL penicillin, 100 μg/mL streptomycin, 0.3 mg/mL Glutamine (PSG, Gibco, Thermo Fisher Scientific, Waltham, MA, USA) and 0.05 nM beta-mercaptoethanol (Merck, Kenilworth, NJ, USA). NK92 Wild Type (WT) cells were obtained from DSMZ (Braunschweig, Germany). NK92 CD16^+^ cells were generated as previously described by inserting CD16a cDNA (CD16a-176V variant (a.k.a. 158V variant when the amino acid enumeration does not include the signal sequence)) into a retroviral expression vector pBMN-IRES-EGFP and subsequently used to stably transduce the cells [25]. Both NK92 cell lines (WT and CD16^+^) were cultured in Minimum Essential Medium Eagle—Alpha Modification (Alpha MEM, Gibco, Thermo Fisher Scientific, Waltham, MA, USA) supplemented with PSG, 12.5% FCS, 12.5% Horse Serum (HS, Gibco, Thermo Fisher Scientific, Waltham, MA, USA) and 80 U/mL interleukin 2 (IL-2, Novartis, Basel, Switzerland). Cell cultures were passaged 2 or 3 times a week and were maintained in an incubator at 37 °C, 95% humidity, 5% CO_2_.

### 2.3. Peripheral Blood Mononuclear Cells and Natural Killer Cell Isolation

Blood samples were obtained from healthy volunteers and patients with CRC under written informed consent at the Amsterdam UMC (location VU University Medical Center, Amsterdam), Amstelland Hospital (Amstelveen), and Netherlands Cancer Institute/Antoni van Leeuwenhoek Hospital (Amsterdam). Patient and healthy donor characteristics are shown in Supplementary Table S1.

Peripheral blood mononuclear cells (PBMC) were isolated using either Lymphoprep^TM^ (STEMCELL Technologies, Vancouver, BC, Canada) or CPT tubes (BD Biosciences, Franklin Lakes, NJ, USA) on a density gradient centrifugation. Natural Killer (NK) cells were isolated from PBMCs through magnetic bead-activated cell sorting using the MACS NK cell Isolation Kit (Miltenyi Biotec, Bergisch Gladbach, Germany) according to the manufacturer’s instructions. NK cell purity was assessed by flow cytometry using CD3 BV711, CD16 BV768 (both BD Horizon, BD Biosciences, Franklin Lakes, NJ, USA), and CD56 APC-Vio770 (Miltenyi Biotec, Bergisch Gladbach, Germany). The NK cell purity was on average 83.5% (SEM: 1.71), and the mean NK cell expression of CD16 was 87.5% (SEM: 1.88).

### 2.4. Flow Cytometry Assessments

To assess binding to EGFR and CD16, both A431 and PBMCs were incubated for 60 min at 4 °C with the bispecific VHHs in a 96-well U-bottom plate at the indicated concentrations. Thereafter, the cells were washed 3 times followed by 30 min of incubation at 4 °C with a FITC labeled anti-llama antibody (Bioke, Leiden, the Netherlands). NK cells in PBMC were gated as CD56^+^ (APC-Vio770 or CD510 (BD Horizon, BD Biosciences, Franklin Lakes, NJ, USA)) and CD3^−^ (BV711 or PE (BD Biosciences, Franklin Lakes, NJ, USA)). Due to the presence of GFP in CD16^+^ NK92, we detect bispecific VHH binding to NK92 (WT or CD16^+^) by incubating the cells for 60 min at 4 °C with the bispecific VHHs previously biotinylated using NHS-D-biotin (Sigma-Aldrich, Saint Louis, MO, USA) according to the manufacturer’s instructions. Afterward, the cells were washed 3 times and stained with streptavidin (APC, Thermo Fisher Scientific, Waltham, MA, USA) for 30 min at 4 °C. Tumor cell lines were analyzed for the expression of multiple ligands of both activating and inhibitory NK cell receptors. Moreover, the expression of EGFR was assessed on tumor cell lines, Epcam^+^CD45^−^ cells in dissociated CRC peritoneal metastatic samples, and on Epcam^dim^CD45^−^ epithelial cells in dissociated normal (non-malignant) peritoneal tissue of the same CRC patients. The following mAbs, conjugated with the listed fluorochromes, were used for cell staining: HLA-E (eBioscience, Thermo Fisher Scientific, Waltham, MA, USA), HLA-G, PVR, MICA/B, (all from Biolegend, San Diego, CA, USA), ULBP2/5/6, ULBP1, ULBP3 (all from R&D systems, Minneapolis, MN, USA), HLA-ABC (Thermo Fisher Scientific, Waltham, MA, USA) all conjugated to PE, and EGFR BV421 (Biolegend, San Diego, CA, USA). Next, the distribution of CD16 on immune cells was analyzed in both PBMC from patients with metastatic CRC and dissociated CRC peritoneal metastatic lesions of patients. For cell staining, the following fluorochrome-conjugated mAbs were used: CD56 APC-Vio770, CD3 BV711, CD16 BV786, CD14 PE-CF594 (BD Biosciences, Franklin Lakes, NJ, USA), γδ TCR BV421 (BD Pharmingen, BD Biosciences, Franklin Lakes, NJ, USA), and CD11b APC (BD Biosciences, Franklin Lakes, NJ, USA). Details on the antibodies’ catalog and clone numbers are shown in Supplementary Table S2. Flow-cytometric measurements were performed using LSRFortessa™ (BD Biosciences, Franklin Lakes, NJ, USA), and the analyses were executed with Kaluza 1.3 (Beckman Coulter, Brea, CA, USA).

### 2.5. Collection and Dissociation of Patient-Derived Tissue Samples

Tissue samples from patients with metastatic CRC undergoing cytoreductive surgery (CRS) and Hyperthermic Intraperitoneal Chemotherapy (HIPEC) were collected from patients during surgery before HIPEC under written informed consent at the Amsterdam UMC (location VU University Medical Center, Amsterdam). Both cancer and normal (non-malignant) tissue were dissociated within 2 h after collection from the patient into a single-cell suspension using a dissociation medium consisting of RPMI, supplemented with 0.1% DNase I (Roche, Basel, Switzerland), 0.14% Collagenase A (Roche, Basel, Switzerland), 5% FCS, and PSG. The specimens were incubated at 37 °C for one to three 45-min dissociation rounds. Thereafter, the cell suspension was run through a 100-μm cell strainer (Corning, NY, USA), erythrocytes were lysed using a shock buffer containing NH_4_Cl (Merck, Kenilworth, NJ, USA), KHCO_3_ (Merck, Kenilworth, NJ, USA), and EDTA (Titriplex III, Merck, Kenilworth, NJ, USA), and the cells were counted. Cells were either used immediately or cryopreserved in liquid nitrogen until further use. Patient characteristics are shown in Supplementary Table S1.

### 2.6. Functional Assays

To explore the functionality of the bispecific VHHs, degranulation and cytotoxicity assays were performed by culturing various cancer cell lines (A431, HCT116, and Colo829) with purified NK cells from healthy donors, either cultured in medium overnight (resting NK cells) or after overnight activation with 1000 U/mL IL-2 (Novartis, Basel, Switzerland) and 10 ng/mL IL-15 (eBioscience, Thermo Fisher Scientific, Waltham, MA, USA), as previously described [26,27] (activated NK cells), or with WT or CD16^+^ NK92 cells. Cultures were performed at 37 °C in a total volume of 200 μL RPMI in a 96-well plate. Supplementation with 80 U/mL IL-2 was applied to the NK92 assays. An effector to target ratio of 1:1 was used. Both bispecific VHHs were used in a concentration of 100 nM unless otherwise indicated. Degranulation was evaluated after 4 h, and it was based on the percentage of CD107a^+^ (PE, eBioscience, Thermo Fisher Scientific, Waltham, MA, USA) NK cells defined as CD45^+^ (AF700, Biolegend, San Diego, CA, USA), CD56^+^ (APC-Vio770), and CD3^−^ (BV711) for peripheral blood NK cells, and as CD56^+^ for NK92. Cytotoxicity was assessed after 24 h, and it was based on the percentage of living tumor cells relative to the tumor-alone control quantified with counting beads (123count eBeads, Invitrogen, Thermo Fisher Scientific, Waltham, MA, USA). Living tumor cells were gated as Epcam^+^ (Biolegend, San Diego, CA, USA), CD45^−^ (AF700), and 7AAD^−^ (Sigma Aldrich, Saint Louis, MO, USA) for A431 and HCT116 or just as CD45^−^ and 7AAD^−^ for Colo829, due to the non-epithelial nature of this cell line.

Assessment of the ability of the C-7 bispecific VHH to activate patient-derived NK cells was performed by degranulation and cytotoxicity assays using overnight-activated monocyte-depleted (through a 2-h plastic adhesion step to eliminate the possibility of a (non-classical/intermediate) monocyte-related effect) PBMC from patients with CRC and the A431 cell line at an effector to target ratio of 4:1. The ability of C-7 to induce NK cell mediated-killing of patient-derived cancer cells was assessed by co-culturing dissociated CRC peritoneal metastatic lesions or normal (non-malignant) peritoneal tissue with autologous PBMC. The cells were co-cultured in a PBMC to tissue cell ratio of 5:1 in a total volume of 200 μL RPMI in a 96-well plate. For the tumor samples, the readout was performed after 1, 3, and 7 days, while, for the healthy tissue, it was performed just at days 1 and 3 due to the loss of healthy epithelial cells upon prolonged in vitro culturing. The readout was based on the absolute numbers of living tumor cells defined as Epcam^+^ (FITC), CD45^−^ (AF700), and 7AAD^−^ or living non-malignant epithelial cells defined as Epcam^dim^ (FITC), CD45^−^ (AF700), and 7AAD^−^ both quantified with counting beads. To assess the possible effects of PBMC-derived CD16^+^ non-classical/intermediate monocytes in our model, similar assays were performed by co-culturing dissociated CRC peritoneal metastatic lesions with autologous PBMC that were depleted of monocytes by magnetic bead-activated cell sorting using CD14 MicroBeads (Miltenyi Biotec, Bergisch Gladbach, Germany) according to manufacturer’s instructions. NK92 CD16^+^ cells were co-cultured with dissociated CRC peritoneal metastatic lesions at an effector to tissue cell ratio of 1:1, 1:5, or 1:10 in a total volume of 200 μL RPMI (without IL-2 supplementation) in a 96-well plate in the presence or absence of C-7. The readout was performed after 1, 3, and 7 days and it was based on the absolute number of living tumor cells defined as Epcam^+^ (FITC), CD45^−^ (AF700), and 7AAD^−^ quantified with counting beads. For all the patient material-related assays, C-7 was used at a concentration of 100 nM.

### 2.7. Cytometric Bead Array

A cytometric bead array (CBA) was performed on supernatants collected after 4 h from co-cultures of overnight-activated healthy donor-derived NK cells and A431 tumor cells in the presence or absence of either 100 nM C-7 or 5 μg/mL cetuximab [28]. The CD8/NK LEGENDplex™ (Biolegend, San Diego, CA, USA) kit containing the following analytes was used according to the manufacturer’s instructions: TNF, sFasL, Granzyme A, Granzyme B, Perforin, Granulysin.

Moreover, a CBA was also performed on the supernatants collected after 24 h from the co-culture of dissociated CRC peritoneal metastatic lesions and autologous PBMC. The IFNγ, TNF, IL-10, IL-6, and CXCL10 Flex Set Kits (BD Biosciences) were used according to the manufacturer’s instructions.

### 2.8. Statistical Analysis

Statistical analyses were performed with GraphPad version 9.1.0 (GraphPad Software, San Diego, CA, USA). The data distribution was tested for normality and guided the selection of the appropriate statistical tests used for analyses. Binding assays over a range of VHH concentrations were analyzed with two-way ANOVA and nonlinear regression analysis to compute the apparent equilibrium dissociation constant (K_d_). Differences in CD16 binding between the bispecific VHHs and the CD16 mAb were assessed with two-tailed paired *t*-tests. For 24-h cytotoxicity assays, 4-h degranulation assays, and CBA (of supernatants from co-cultures of healthy donor NK cells and A431 tumor cells), the *p*-values were calculated with two-tailed paired *t*-test or, when multiple bispecific VHH concentrations were tested, with a two-way ANOVA analysis, followed by Bonferroni’s multiple comparison analysis and nonlinear regression analysis to assess the EC50 values. The Mann–Whitney test was applied to compare the EGFR expression on tumor and epithelial cells. Two-way ANOVA with Dunnett’s multiple comparisons analysis was used to analyze differences for the 3-day survival assay with normal (non-malignant) peritoneal tissue and all the 7-day survival assays, apart from the monocyte-depleted PBMC test for which Tukey’s multiple comparisons test was used. Finally, one-way ANOVA with Dunnett’s multiple comparison test was used to analyze the CBA performed on supernatants of co-cultures of patient tumor and autologous PBMC samples. Due to the non-normal distribution of some of the CBA results, Friedman’s ANOVA with Dunn’s multiple comparison analysis was also performed. Findings were considered significant when *p*-values were <0.05.

## 3. Results

### 3.1. Comparison of N- and C-Terminal Positioning of the Individual VHHs in the CD16-EGFR Bispecific VHH

**Figure 1:**
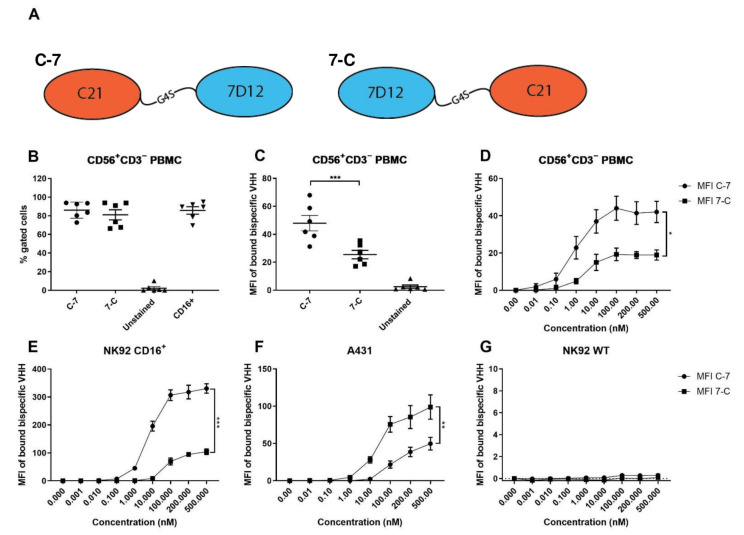
Binding characteristics of C-7 and 7-C bispecific VHHs to CD16 and EGFR. (**A**) Graphical representation of the bispecific VHHs. (**B**) Percentage of bispecific VHH^+^ cells (at 100 nM) and CD16^+^ cells among CD56^+^CD3^−^ cells in PBMC, *n* = 6. (**C**) Median Fluorescence Intensity (MFI) of bound bispecific VHH on CD56^+^CD3^−^ cells in PBMC at 100 nM, *n* = 6. (**D**) MFI of bound bispecific VHH on CD56^+^CD3^−^ cells in PBMC *n* = 3. (**E**) MFI of bound bispecific VHH to CD16^+^ NK92 *n* = 3. (**F**) MFI of bound bispecific VHH to A431 (EGFR^++^) *n* = 8; (**G**) MFI of bound bispecific VHH to CD16^−^EGFR^−^ NK92 WT, *n* = 3. The data are presented as mean ± SEM. Significance is presented as *p* < 0.05 *, <0.01 **, <0.001 ***. *p*-values were determined by two-tailed paired *t*-test (**B**,**C**) or two-way ANOVA (**D**,**G**). Abbreviations: PBMC = peripheral blood mononuclear cells, C-7 = C21-7D12 bispecific VHH, 7-C = 7D12-C21 bispecific VHH.

Bispecific VHHs targeting both CD16 and EGFR were generated by recombinant fusing of the CD16-specific VHH C21 to the EGFR-specific VHH 7D12 using a Gly4Ser-linker [14,15]. The effect of N- and C-terminal positioning of the individual VHHs in the bispecific VHH was assessed by producing two bispecific VHHs: C21-7D12 (C-7) and 7D12-C21 (7-C) (Figure 1A).

First, we assessed whether C-7 and 7-C could be used to identify CD16 expression on CD56^+^CD3^−^ NK cells in PBMC. For this, the binding of both bispecific VHHs at 100 nM, detected with FITC-labeled anti-llama polyclonal antibodies, was compared to the binding of a commercially available CD16 mouse anti-human mAb labeled with BV786 (clone 3G8, BD Horizon, BD Biosciences, Franklin Lakes, NJ, USA). No statistically significant differences in the percentage of positive cells between C-7 and 7-C were found compared to the CD16 BV786 staining (*p* = 0.83 and *p* = 0.33, respectively) (Figure 1B and Figure S1), indicating that the bispecific VHHs, similarly to a commercially available CD16 mAb, can reliably be used to detect CD16 on NK cells. However, 7-C showed significantly lower median fluorescence intensity (MFI) (*p* = 0.004, Figure 1C) compared to C-7, indicating a lower binding capacity of 7-C to CD16. The apparent affinity of the two bispecific VHHs to CD16, expressed by either healthy donor PBMC-derived CD56^+^CD3^−^ NK cells or by the NK92 cell line transfected to express CD16, and to EGFR expressed by the EGFR^++^ A431 cell line, was assessed using a concentration range of the constructs. Both orientations of the bispecific VHH were able to bind to both CD16 and EGFR. C-7 was found to have significantly higher binding affinity to CD16 as assessed using both CD56^+^CD3^−^ PBMC (*p* = 0.034) and CD16^+^ NK92 (*p* = 0.0004) (Figure 1D,E). In contrast, 7-C was found to have significantly higher binding affinity to EGFR (*p* = 0.002) (Figure 1F). Details on the apparent K*_d_* can be found in Table 1. Binding specificity was also tested by analyzing binding to the CD16^−^EGFR^−^ NK92 WT cell line. No binding was found, confirming the specificity of the bispecific VHHs to both EGFR and CD16 (Figure 1G).

Apparent equilibrium dissociation constant (K_d_) of C-7 and 7-D binding to both CD16 on CD56^+^CD3^−^ NK cells or NK16^+^ NK92 and EGFR on the A431 tumor cell line. The K_d_s were calculated using nonlinear regression analysis.

### 3.2. CD16-EGFR Bispecific VHHs Trigger Equivalent NK Cell Degranulation and Cytotoxicity against EGFR Expressing Targets

**Figure 2:**
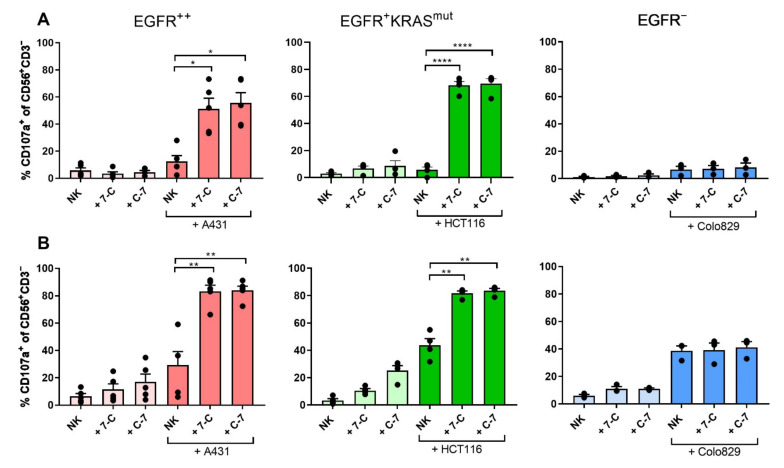
CD16-EGFR bispecific VHHs induce degranulation of NK cells in the presence of EGFR-expressing tumor cells. Degranulation of healthy donor-derived NK cells after a 4-h co-culture ±100 nM bispecific VHHs ± tumor cell lines expressing different levels of EGFR: A431 (EGFR^++^), HCT116 (EGFR^+^RAS^mut^), Colo829 (EGFR^−^). (**A**) Resting NK cells were used as effectors; (**B**) NK cells were pre-activated overnight with IL-2 and IL-15. Degranulation was determined by assessing the percentage of CD107a^+^ cells. E:T ratio: 1:1. The bars represent mean ± SEM. Significance is presented as *p* < 0.05 *, <0.01 **, <0.0001 ****. A431 *n* = 5 (**A**,**B**); HCT116 *n* = 4 (**A**,**B**); Colo829 *n* = 3 (**A**,**B**). *p*-values were determined by two-tailed paired *t*-test. Abbreviations: C-7 = C21-7D12 bispecific VHH, 7-C = 7D12-C21 bispecific VHH.

Next, the ability of both bispecific VHHs to induce NK cell degranulation, assessed as percentage of CD107a^+^ cells in CD56^+^CD3^−^ NK cells, and to trigger tumor cell killing, determined as the percentage of living tumor cells relative to the tumor-alone condition, was investigated by co-culturing healthy donor-derived NK cells, purified using magnetic bead-activated cell sorting, with tumor cell lines with different levels of EGFR expression and either wild-type or mutant for KRAS: A431 (EGFR^++^), HCT116 (EGFR^+^KRAS^mut^), Colo829 (EGFR^−^) (Supplementary Figure S2A) in the presence or absence of 100 nM of either of the bispecific VHHs. The NK cells used for these experiments were either cultured in medium overnight (resting NK cells) or pre-activated overnight using IL-2 and IL-15 (activated NK cells). In co-cultures with EGFR^+^ tumor cell lines, the levels of degranulation of resting healthy donor-derived NK cells were significantly higher in the presence of either of the bispecific VHHs (C-7 *p* = 0.016; 7-C *p* = 0.025 when using A431 as target cells; or C-7 *p* < 0.0001; 7-C *p* < 0.0001 when using HCT116 as target cells) (Figure 2A). Similarly, the bispecific VHHs triggered stronger degranulation of activated healthy donor-derived NK cells co-cultured in the presence of A431 (C-7 *p* = 0.002; 7-C *p* = 0.001) and HCT116 (C-7 *p* = 0.004; 7-C *p* = 0.006) (Figure 2B).

**Figure 3:**
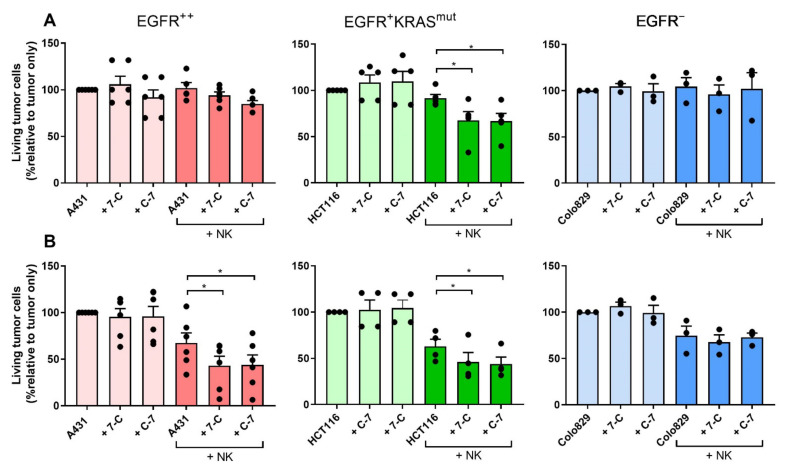
CD16-EGFR bispecific VHHs induce lysis of EGFR expressing tumor cells in the presence of NK cells. Cytotoxicity exerted by healthy donor-derived NK cells after a 24-h co-culture ±100 nM bispecific VHHs ± tumor cell lines expressing different levels of EGFR: A431 (EGFR^++^), HCT116 (EGFR^+^RAS^mut^), Colo829 (EGFR^−^). (**A**) Resting NK cells were used as effectors; (**B**) NK cells were pre-activated overnight with IL-2 and IL-15. Cytotoxicity was determined by assessing the relative percentage of living tumor cells compared to the tumor-alone condition. E:T ratio: 1:1. The bars represent mean ± SEM. Significance is presented as *p* < 0.05 *. A431 *n* = 6 (**A**,**B**); HCT116 *n* = 5 (**A**) and *n* = 4 (**B**); Colo829 *n* = 3 (**A**,**B**). *p*-values were determined by a two-tailed paired *t*-test. Abbreviations: C-7 = C21-7D12 bispecific VHH, 7-C = 7D12-C21 bispecific VHH.

Although the bispecific VHHs did not trigger tumor cell cytotoxicity when resting healthy donor-derived NK cells were co-cultured with A431 tumor cells (C-7 *p* = 0.13; 7-C *p* = 0.34) (Figure 3A), both bispecific VHHs did enhance cytotoxicity of A431 tumor cells by activated healthy donor-derived NK cells (C-7 *p* = 0.049; 7-C *p* = 0.020) (Figure 3B). This difference is possibly due to a certain level of resistance of A431 to NK cell-mediated killing which is overcome when NK cells are activated using IL-2 and IL-15. Both bispecific VHHs did trigger cytolysis of HCT116, a KRAS^mut^ cell line, by either resting (C-7 *p* = 0.015; 7-C *p* = 0.042) or activated (C-7 *p* = 0.022; 7-C *p* = 0.046) healthy donor-derived NK cells (Figure 3A,B), indicating that the presence of a KRAS mutation does not hamper the activity of the bispecific VHHs. As expected, when NK cells (either resting or activated) were co-cultured with the EGFR^−^ Colo829 tumor cell line, no NK cell degranulation nor tumor cell cytotoxicity was induced by the bispecific VHHs (*p* > 0.05) (Figure 2 and Figure 3). Of note, while the bispecific VHH induced more pronounced tumor cell lysis when overnight activated NK cells were used, this also increased the “spontaneous” (i.e., in the absence of the bispecific VHH) cytolysis of tumor cells and likely reflects the enhanced NK cell activation state reached after cytokine stimulation. To explore whether the difference in sensitivity to NK cell-mediated killing of the cell lines A431 and HCT116 was related to differences in the expression of various ligands to NK cell activating (NKG2D, DNAM1) or inhibitory (KIR2D, NKG2A) receptors, expression levels of these ligands was assessed on both tumor cell lines (Supplementary Figure S2B). No clear relation between the NK cell receptors and their respective ligands on the tumor cell lines was found, suggesting that other factors determine the observed difference in sensitivity to NK cell lysis between these tumor cell lines.

**Figure 4:**
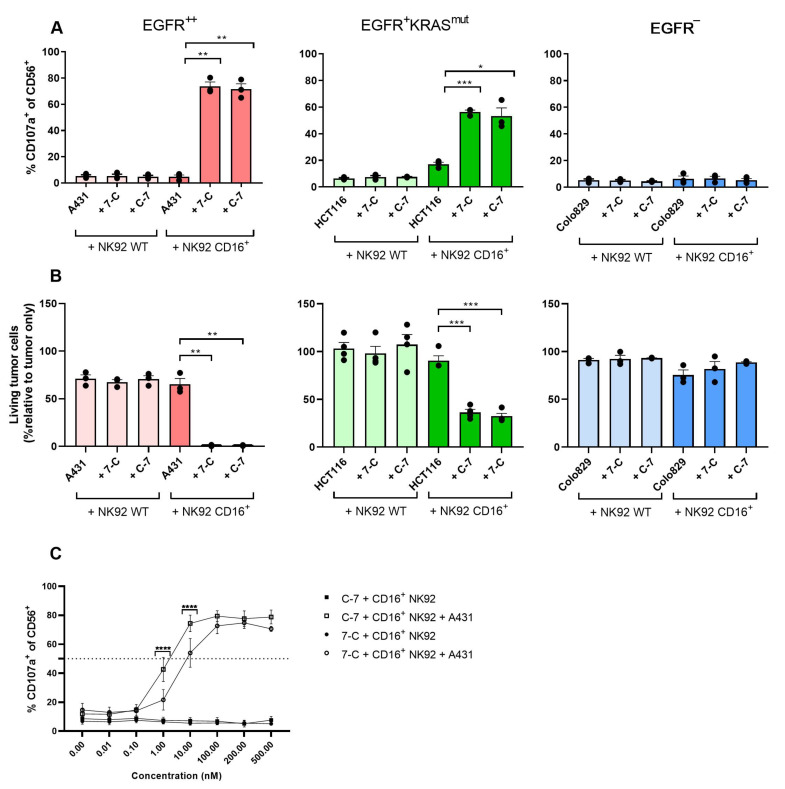
CD16-EGFR bispecific VHHs induce degranulation of CD16^+^ NK92 cells and cytotoxicity against EGFR-expressing tumor cells. Degranulation (**A**) and cytotoxicity (**B**) of NK92 WT and NK92 CD16^+^ co-cultured ±100 nM bispecific VHHs ± tumor cell lines expressing different levels of EGFR: A431 (EGFR^++^), HCT116 (EGFR^+^RAS^mut^), Colo829 (EGFR^−^). Degranulation was determined after 4 h by assessing the percentage of CD107a^+^ cells. Cytotoxicity was determined after 24 h by assessing the relative percentage of living tumor cells compared to the tumor-alone condition. E:T ratio: 1:1. The bars represent mean ± SEM. A431 *n* = 3 (**A**,**B**); HCT116 *n* = 3 (**A**) and *n* = 4 (**B**); Colo829 *n* = 3 (**A**,**B**). (**C**) Degranulation of NK92 CD16^+^ after 4-h co-culture with A431 ± concentration range of the bispecific VHHs. Degranulation was assessed by determining the percentage of CD107a^+^ cells. E:T ratio: 1:1. The data are presented as mean ± SEM. *n* = 4. Significance is presented as *p* < 0.05 *, <0.01 **, <0.001 ***, <0.0001 ****. *p*-values were determined by two-tailed paired *t*-test (**A**,**B**) or with two-way ANOVA with Bonferroni multiple comparison analysis (C). Abbreviations: C-7 = C21-7D12 bispecific VHH, 7-C = 7D12-C21 bispecific VHH.

To further confirm that the activity of the bispecific VHHs depended on binding to both CD16 and EGFR the A431, HCT116, and Colo829 cell lines were co-cultured with the WT (CD16^−^) or CD16-transfected NK cell line NK92. The bispecific VHHs only induced statistically significant degranulation of NK cells and lysis of tumor cells when tumor cells expressed EGFR and NK92 cells expressed CD16 (Figure 4A,B). Although the ability of the bispecific VHHs to induce NK92 CD16^+^ degranulation against the EGFR^+^^+^ A431 cell lines was very similar, C-7 was found to trigger stronger NK cell degranulation at lower concentrations (*p* < 0.0001, at 10 nM; *p* < 0.0001 at 1 nM) compared to 7-C leading to a lower EC50 (C-7: 1.15 nM (95%CI 0.66;2.03); 7-C: 5.20 nM (95%CI 2.34;11.16)) (Figure 4C). For this reason and because of its superior binding affinity to CD16, C-7 was chosen for further experimental testing.

### 3.3. The Bispecific C-7 VHH and the Anti-EGFR mAb Cetuximab Trigger Similar NK Cell Secretion of Cytotoxic Mediators

**Figure 5:**
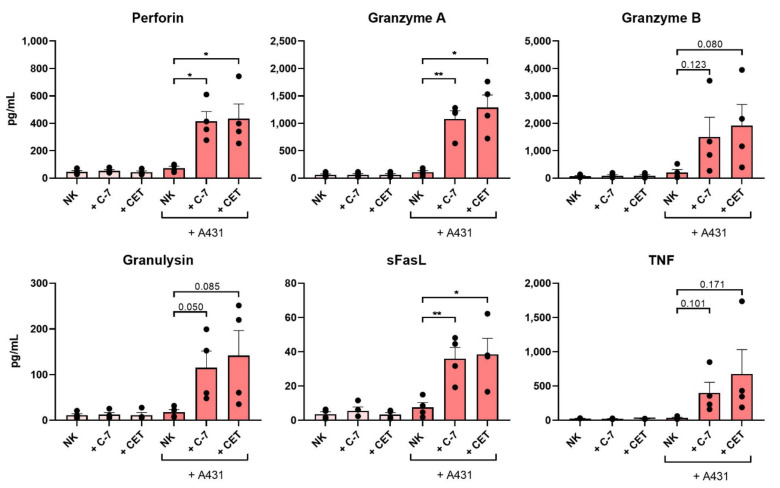
The C-7 bispecific VHH and cetuximab trigger NK cell secretion of pro-inflammatory and cytotoxic mediators. Cytometry Bead Array performed on supernatant of overnight-activated healthy donor-derived NK cells ± A431 ± 100 nM bispecific VHHs ±5 μg/mL cetuximab. E:T ratio 1:1. *n* = 4. The data are presented as mean ± SEM. Significance is indicated or presented as *p* < 0.05 *, <0.01 **. *p*-values are determined by two-tailed paired t-test. Abbreviations: C-7 = C21-7D12 bispecific VHH, CET = cetuximab.

To assess whether C-7 would trigger NK cells to release molecules involved in the cytotoxic pathways reported for NK cells, overnight-activated healthy donor-derived NK cells, purified using magnetic bead-activated cell sorting, were co-cultured with the EGFR^++^ A431 cell line in the presence or absence of either 100 nM C-7 or 5 μg/mL cetuximab. C-7 and cetuximab similarly triggered the release of perforin (C-7 *p* = 0.011, cetuximab *p* = 0.035), granzyme A (C-7 *p* = 0.005, cetuximab *p* = 0.012), and soluble Fas ligand (sFasL, C-7 *p* = 0.008, cetuximab *p* = 0.034) (Figure 5). Moreover, although not statistically significant, higher levels of granzyme B (C-7 *p* = 0.123, cetuximab *p* = 0.080), granulysin (C-7 *p* = 0.050, cetuximab *p* = 0.085), and TNF (C-7 *p* = 0.101, cetuximab *p* = 0.170) (Figure 5) were found in the presence of both C-7 and cetuximab. The pattern of secretion of cytotoxic and inflammatory molecules were strikingly similar between C-7 and cetuximab, thereby suggesting that the C-7 bispecific VHH triggers the same major NK cell cytotoxic pathways as those involved in IgG_1_-mediated ADCC.

### 3.4. NK Cells in PBMC of Metastatic Colorectal Cancer Patients Can Be Activated to Lyse Tumor Cells by the Bispecific C-7 VHH

**Figure 6:**
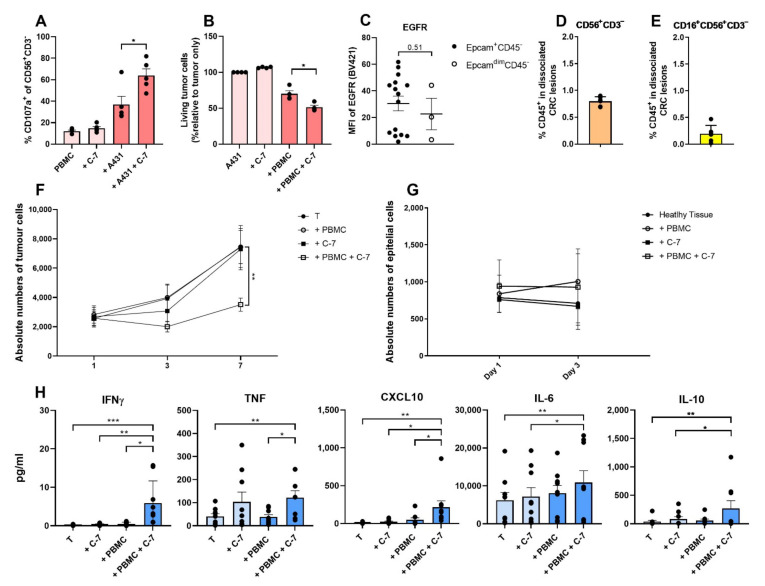
Activity of the bispecific C-7 VHH using metastatic CRC patient-derived PBMC and dissociated tumor samples. (**A**) Degranulation of NK cells (defined as CD45^+^CD56^+^CD3^−^ cells) and (**B**) cytotoxicity of A431 tumor cells after a 4-h (degranulation) and 24-h (cytotoxicity) co-culture of monocyte-depleted PBMC derived from patients with stage II/III CRC pre-activated overnight with IL-2 and IL-15 ± A431 (EGFR^++^) ±100 nM C-7. E:T ratio 4:1. Degranulation was determined by assessing the percentage of NK cells expressing CD107a^+^. Cytotoxicity was determined by assessing the relative percentage of living tumor cells compared to the tumor-alone control. *n* = 5 (**A**), *n* = 4 (**B**). (**C**) EGFR MFI of Epcam^+^CD45^−^ tumor cells in dissociated CRC peritoneal metastatic lesions (*n* = 15) and Epcam^dim^CD45^−^ epithelial cells, in (non-malignant) peritoneal tissue (*n* = 3). CD56^+^CD3^−^ (**D**) and CD16^+^CD56^+^CD3^−^ (**E**) percentage of CD45^+^ cells in dissociated CRC peritoneal metastatic lesions, *n* = 6. (**F**) Absolute number of Epcam^+^CD45^−^ tumor cells from dissociated CRC peritoneal metastatic lesions cultured ± autologous PBMC (E:T ratio 5:1) ±100 nM C-7 for 1, 3, and 7 days. *n* = 8. The significance level refers to the conditions: “T+PBMC” versus “T+PBMC+C-7”. (**G**) Absolute number of Epcam^dim^CD45^−^ cells from dissociated normal (non-malignant) peritoneal tissue of patients with metastatic CRC ± autologous PBMC (E:T ratio 5:1) ±100 nM C-7, incubated for 1 and 3 days. *n* = 3. (**H**) Cytometry Bead Array performed on supernatant of dissociated CRC peritoneal metastases cultured for 24 h ± autologous PBMC (E:T ratio 5:1) ± 100 nM C-7. *n* = 9. The data are presented as mean ± SEM. Significance is presented as *p* < 0.05 *, <0.01 **, <0.001 ***. No *p*-values are mentioned in case *p* > 0.05. *p*-values are determined by two-tailed paired *t*-test (**A**,**B**); Mann–Whitney test (**C**); two-way ANOVA with Dunnett’s multiple comparison test (**F**,**G**); one-way ANOVA with Dunnett’s multiple comparison test (H: IL-6); Friedman test with Dunn’s multiple comparison test (**H**: IFNγ, TNF, CXCL10, IL-10). Abbreviations: T = tumor, C-7 = C21-7D12 bispecific VHH, PBMC = peripheral blood mononuclear cells.

NK cells from patients with cancer can be functionally impaired and as a result can lack tumor cell killing ability [29,30]. Because of this, the ability of C-7 to enhance the function of patient-derived NK cells was tested using PBMC from patients with stage II/III CRC cultured in the presence of the EGFR^++^ A431 cell line. Details on patient characteristics can be found in Supplementary Table S1. PBMC used for these experiments were pre-activated overnight with IL-2 and IL-15, to facilitate detection of a bispecific VHH mediated effect, and depleted of (CD16^+^) non-classical/intermediate monocytes through an adhesion step to allow for a more specific assessment of the cytotoxic potential of CD16^+^ NK cells in patient PBMC. Though some degranulation of NK cells, defined as the percentage of CD107a^+^ cells in CD56^+^CD3^−^ cells, was observed after a 4-h co-culture of PBMC and A431 tumor cells alone, NK cell degranulation was significantly increased when C-7 was added to these cultures (*p* = 0.026) (Figure 6A). As expected, C-7 did not trigger NK cell degranulation in the absence of EGFR expressing tumor target cells. In line with these data, although the addition of monocyte-depleted PBMC to A431 tumor cells already resulted in a decrease in viable tumor cells after 24 h, this cytolytic effect was potentiated by C-7 (*p* = 0.031) (Figure 6B). In the timeframe of this experiment no increase in degranulation of CD3^+^ T cells was observed (*p* = 0.221; Supplementary Figure S3). These results indicate that C-7 is able to induce activation (degranulation) and enhance the cytolytic activity of NK cells in PBMC of colorectal cancer patients.

### 3.5. The Bispecific C-7 VHH Triggers Cytokine and Chemokine Production and Controls Tumor Growth in Co-Cultures of Patient-Derived Metastatic CRC Single-Cell Suspensions and Autologous PBMC

Next, we assessed the NK cell activity enhancing effect of the bispecific C-7 VHH using enzymatically dissociated tumor samples derived from patients with peritoneal CRC metastases scheduled for cytoreductive surgery (CRS) and HIPEC. Details on patient characteristics can be found in Supplementary Table S1. Patient CRC cells, defined as Epcam^+^CD45^−^ cells, expressed variable levels of EGFR (Figure 6C). As the intratumoral frequency of NK cells in metastatic CRC tissue was reported to be significantly lower than in healthy tissue [31], the presence of NK cells, particularly CD16^+^ NK cells, was analyzed in these dissociated tumor samples. Because the frequency of NK cells, defined as CD45^+^CD56^+^CD3^−^ cells, and CD16^+^ NK cells was indeed low (0.80% (SEM 0.03) and 0.19% (SEM: 0.06) of total CD45^+^ leukocytes, respectively) (Figure 6D,E), we tested the activity of C-7 not only in 7-day cultures of patient-derived dissociated CRC tumor cells (not selected for specific EGFR expression levels) alone but also in co-cultures of CRC tumor cells and autologous patient-derived PBMC. An example of the gating strategy used to analyze the cytotoxicity assays can be found in Supplementary Figure S4. Tumor growth control was not observed when C-7 was added to 7 day-cultures of dissociated CRC tumor samples alone; however, when C-7 was added to co-cultures of dissociated CRC samples and autologous PBMC, significant tumor growth inhibition was observed (*p* = 0.002) (Figure 6F). Interestingly, no C-7 induced lysis of epithelial cells, defined as Epcam^dim^CD45^−^, was observed when patient-derived normal (non-malignant) peritoneal tissue samples were cultured for up to 3 days with C-7, regardless of the addition of autologous PBMC, demonstrating the tumor-specific nature of the NK cell response (*p* > 0.05) (Figure 6G). Of note, the level of EGFR expressed by epithelial cells was not statistically significantly different compared to that expressed by tumor cells (*p* = 0.51) (Figure 6C), suggesting additional factors to be involved in modulating NK cell cytolytic activity.

The impact of C-7 on the levels of various cytokines (IFNγ, TNF, IL-6, and IL-10) and the chemokine CXCL10 was assessed in 1-day co-culture supernatants of dissociated CRC tumor cells and autologous PBMC (Figure 6H). Higher levels of IFNγ and TNF, two cytokines produced by activated NK and T cells [5,32,33,34], were found in the presence of both C-7 and autologous PBMC, suggesting predominant production by peripheral blood NK cell and/or (CD16^+^ or indirectly CD16^−^) T cells. Of interest, in the absence of PBMC, C-7 also increased TNF levels, although this did not meet statistical significance. As macrophages (defined as CD14^+^CD11b^+^) constitute a major TNF-producing cell subpopulation [32,35] and were the dominant CD16^+^ cell subset (Supplementary Figure S5) in the CRC tumor suspensions, the observed increase in TNF levels could be macrophage-derived. Enhanced production of CXCL10, an important inducer of immune effector cell migration [36,37,38], was also observed when both PBMC and C-7 were co-cultured with dissociated CRC samples. IL-10 and IL-6 are highly pleiotropic cytokines that are mostly known for their pro-tumorigenic and immune-suppressive effects [39,40], but they can also contribute to T and NK cell activation [39,40,41]. In the presence of both C-7 and autologous PBMC, increased levels of IL-10 and IL-6 were found compared to the tumor alone (*p* = 0.002 and *p* = 0.004, respectively) and the “tumor + C-7” conditions (*p* = 0.032 and *p* = 0.025, respectively). Interestingly, relatively high levels of IL-6 were already present in the tumor control conditions and were further enhanced by the combination of C-7 and autologous PBMC, which might be related to either an increased release from tumor cells upon their lysis and/or Fc-dependent macrophage activation [42].

### 3.6. Non-Classical/Intermediate Monocytes Do Not Affect Bispecific C-7 VHH Mediated Tumor Growth Control in Co-Cultures of Metastatic CRC Single-Cell Suspensions and Autologous PBMC

**Figure 7:**
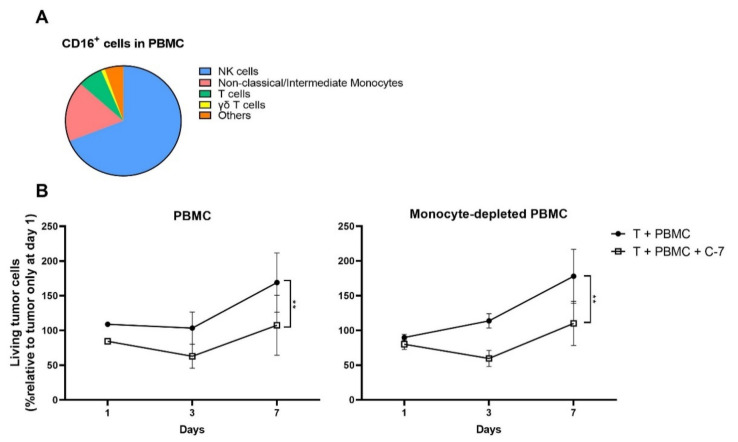
Tumor growth control mediated by the bispecific C-7 VHH in co-cultures of metastatic CRC cells and autologous PBMC is independent of effects on non-classical/intermediate monocytes. (**A**) Distribution of CD16^+^ cells in PBMC from patients with peritoneal CRC metastasis. *n* = 7. (**B**) Dissociated CRC peritoneal metastatic lesions ±100 nM C-7 ± autologous PBMC or monocyte depleted autologous PBMC. E:T ratio: 5:1. *n* = 4. Incubated for 1, 3, and 7 days. Data are presented as mean ± SEM. Significance is presented as *p* < 0.01 **. *p*-values were determined by two-way ANOVA with Tukey’s multiple comparison test (**B**). Abbreviations: T = tumor, C-7 = C21-7D12 bispecific VHH.

To further confirm that the bispecific C-7 VHH-mediated tumor control that was observed in co-cultures of dissociated CRC samples and autologous PBMC was mediated predominantly by CD16^+^ NK cells, we assessed the distribution of CD16 expressing immune cell subsets present in PBMC of patients with peritoneal CRC metastasis. Of all CD16^+^ cells in PBMC, NK cells (CD56^+^CD3^−^) represented the largest CD16^+^ PBMC population (69.3% (SEM 6.30)), while non-classical/intermediate monocytes (CD14^+^CD16^+^) made up the second-largest CD16^+^ PBMC fraction (17.8% (SEM 6.05)), followed by several relatively low-frequency CD16 expressing T cell subsets (Figure 7A). As non-classical/intermediate monocytes represented the predominant CD16^+^ cell population after NK cells in PBMC, we determined whether depletion of monocytes from PBMC using a negative CD14 magnetic bead-mediated selection impacted tumor growth inhibition. In the presence of C-7, similar tumor growth control inhibition was observed when dissociated CRC samples were co-cultured with either monocyte-depleted or non-monocyte-depleted autologous PBMC (*p* = 0.997), indicating that the activity of C-7 is not dependent on non-classical/intermediate monocytes (Figure 7B). Of note, the CD14 magnetic bead-mediated depletion resulted in efficient depletion of both (CD14^dim^CD16^high^) non-classical and (CD14^high^CD16^dim^) intermediate monocytes (Supplementary Figure S6) [43] with an overall efficiency of 96.3% (SEM 1.07).

### 3.7. The Bispecific C-7 VHH Enhances Antitumor Activity of CD16^+^ NK92 Cells against Patient Metastatic CRC Cells

**Figure 8:**
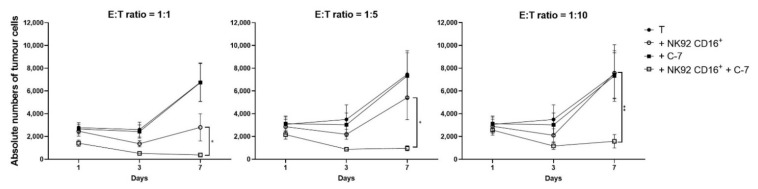
The bispecific C-7 VHH triggers CD16^+^ NK92 cells to control tumor growth using patient metastatic CRC cells. Dissociated CRC peritoneal metastatic samples ± NK92 CD16^+^ ±100 nM C-7 were cultured for 1, 3, and 7 days at multiple E:T ratios, i.e., 1:1 *n* = 6, 1:5 *n* = 5, 1:10 *n* = 5. The significance levels refer to the conditions: “T+NK92 CD16^+^” versus “T+NK92 CD16^+^+C-7”. Data are presented as mean ± SEM. Significance is presented as *p* < 0.05 *, <0.01 **. *p*-values were determined by two-way ANOVA with Dunnett’s multiple comparison analysis. Abbreviations: T = tumor, C-7 = C21-7D12 bispecific VHH.

Despite the known impairment of NK cell functionality in cancer patient PBMC [29,30], the bispecific C-7 VHH was able to activate them and simultaneously control tumor growth. As allogeneic NK cells were reported to have superior antitumor activity compared to autologous NK cells [6], we explored whether the bispecific C-7 VHH could also enhance the antitumor efficacy of allogeneic NK cells. To test this, CD16^+^ NK92 cells were cultured for 1, 3, and 7 days with dissociated patient CRC cells, derived from peritoneal metastases, in the presence or absence of C-7. Multiple CD16^+^ NK92 to tumor cell ratios were tested (Figure 8). In the absence of the bispecific VHH, CD16^+^ NK92 cells could limit tumor growth only at the relatively high 1:1 E:T ratio (Figure 8, left panel). Stronger tumor growth control was noted in the presence of C-7 resulting in statistically significant tumor growth inhibition at E:T ratios as low as 1:10 (*p*: ratio 1:1 = 0.048, ratio 1:5 = 0.023, ratio 1:10 = 0.007) (Figure 8).

## 4. Discussion

NK cells are innate lymphocytes with the ability to recognize and kill cancer cells [1,2]. They can play a role in the immune response against cancer and patients with activated NK cells were found to have a better prognosis [44,45]. However, NK cell activity can be compromised by cancer, as well as cancer therapies, increasing the need for strategies to enhance NK cell functions [6,46]. Here, we report the generation and the functional characterization of a novel EGFR-CD16 bispecific VHH that, in in vitro and ex vivo studies, enhances NK cell function to improve tumor control through simultaneous targeting of CD16 on effector NK cells and EGFR on epithelial cancers. *n*- and C-terminal positioning of the VHHs were compared for binding affinity and function. Both bispecific VHHs induced EGFR specific activation of CD16^+^ NK cells and tumor cell lysis regardless of KRAS tumor mutation status. However, due to its more potent induction of NK cell degranulation (i.e., lower EC50) and stronger binding to CD16, the bispecific C-7 VHH (with the CD16-specific VHH C21 at the N-terminal and the EGFR-specific VHH 7D12 at the C-terminal position) was selected for further experiments. The bispecific C-7 VHH enhanced the activity of patient-derived NK cells and controlled tumor growth in co-cultures of single-cell suspensions derived from peritoneal CRC metastases and autologous PBMC. Although the bispecific C-7 VHH could also potentially trigger the activity of CD16^+^ non-NK cells, such as non-classical/intermediate monocytes that constituted the second-largest CD16^+^ immune cell subset in metastatic CRC patient PBMC, we found that the dominant antitumor effect was mediated via the activation of CD16^+^ NK cells as depletion of non-classical/intermediate monocytes did not affect the tumor growth control triggered by C-7. In the presence of C-7 increased levels of the pro-inflammatory cytokines, IFNy and TNF were detected in supernatants of co-cultures of dissociated patient-derived metastatic CRC samples and autologous PBMC, likely produced by activated CD16^+^ NK cells [5], with a potential contribution of CD16^+^ γδ-T and CD8^+^ T cells [33,34]. Simultaneously we found increased levels of CXCL10, a strong inducer of CD8^+^ T and NK cell migration [36,37,38], which was most likely related to this NK or type-1 T cell activation. Indeed, the increase in CXCL10 correlated closely to the levels of IFNγ (Pearson r: 0.84, *p* < 0.0001), which is in line with previous studies showing CXCL10 production to be IFNγ-induced [47,48]. The increased release of CXCL10 at the tumor site upon C-7-mediated NK cell activation may lead to further attraction of immune effector cells to the tumor microenvironment and might, thus, potentiate other immunotherapeutic interventions, e.g., through immune checkpoint blockade. Increased levels of the anti-inflammatory cytokines IL-6 and IL-10 were also noted in co-cultures of single-cell tumor suspensions with PBMC and C-7. While we did not determine the cellular source, it is known that these cytokines can be produced by a variety of cells [39,40], including activated NK cells [49,50], and, perhaps more likely in our experiments, CD16^+^ monocytes and macrophages that constituted the second most frequent population of CD16^+^ cells in patient PBMC and the dominant fraction in the patient peritoneal CRC metastases [42,51,52]. Although both IL-10 and IL-6 are mostly known for their pro-tumorigenic and immune-suppressive activity, they can also promote immune function via, e.g., the recruitment of effector T cells and enhancement of NK cell cytotoxic activity [39,40].

As NK cells from patients with cancer can be functionally impaired [29,30], various approaches to overcome this hurdle are currently explored. Of those, adoptive transfer of allogeneic NK cells represents an attractive strategy due to its potential to kill tumor cells without the restriction of patient tumor HLA expression [53,54] in the absence of GvHD [7,8,9]. However, allogeneic NK cell transfer was found to be relatively ineffective in solid tumors [6], and strategies to enhance targeted NK cell activity might, therefore, be beneficial. Here, we explored whether tumor growth inhibition could be enhanced by the combined administration of the NK92 cell line, currently in clinical development [55,56,57], transduced to express CD16 and the bispecific C-7 VHH in co-cultures with single-cell suspensions derived from peritoneal metastatic CRC. While administration of CD16^+^ NK92 cells alone controlled tumor growth of metastatic CRC cells at effector to target cell ratios of 1:1, antitumor activity was further enhanced by C-7. At lower effector to target ratios, where no statistically significant tumor growth inhibition was noted from the addition of CD16^+^ NK92 cells alone, C-7 resulted in a strong enhancement of antitumor activity, suggesting that the combination of transfer of allogeneic NK cells and NK cell engaging bispecific antibodies could be of interest and could facilitate recruitment of NK cells to the tumor microenvironment directly, as well as via the production of CXCL10. Moreover, the latter might serve to attract T cells, further facilitating (for instance) effective immune checkpoint blockade, suggesting a further possibility for combination therapies.

Although, the C-7 bispecific VHH and cetuximab triggered a similar release pattern of TNF and cytotoxic molecules by NK cells, the use of a CD16 specific VHH provides several advantages compared to ADCC mediated through binding of the Fc domain of a therapeutic tumor-targeting antibody to CD16. First, CD16 polymorphisms, in particular the presence of a phenylalanine (F) instead of a valine (V) in position 176, can influence the ability of conventional IgG_1_ to induce NK cell-mediated ADCC [58,59,60]. As the C21 VHH has a different CD16 binding site than IgG_1_ antibodies, these polymorphisms will not impact the activity of the bispecific C-7 VHH [14]. Second, conventional IgG_1_-based antibodies will also potentially bind to inhibitory Fc receptors, such as CD32b, expressed by B cells and myeloid cells, which may negatively modulate ADCC mediated through CD16 [17,61], thereby hampering the therapeutic effect of IgG_1_-based antibodies [14,61]. Third, targeting specifically CD16 using our bispecific VHH limits neutrophil activation, as this requires the co-engagement of CD32a and CD16b [62,63]. This leads to a more NK cell-specific effect and minimizes the chances of neutrophil activation, which, in tumor-conditioned microenvironments, would most often exert immune suppressive effects [64]. Of note, although neutrophil activation by C-7 is unlikely, neutrophils might still limit C-7 in vivo activity by behaving as peripheral sink. Finally, VHHs can be produced in a highly cost- and time-efficient manner and, due to their smaller size, have more rapid and more homogeneous tissue penetration compared to conventional antibodies [12].

In addition to the C-7 bispecific VHH, various other EGFR-CD16 bispecific antibodies have recently been reported. These include a similarly structured bispecific VHH, that triggered NK cell degranulation and IFN-γ release but has not yet formally demonstrated actual tumor cell lysis [65], an anti-EGFR x anti-CD16 bispecific VHH cyclobody (Ex16), where both the N and C termini of the VHHs are linked through split-intein circular ligation to protect the compound from proteolysis, but which also negatively impacts its cytotoxic potential [66], and AFM24, an IgG_1_-scFv fusion antibody [67], that, due to its relatively large size (~200 kD versus ~30 kD for the C-7 bispecific VHH), may have reduced potential to deeply penetrate tumor tissue. In this study, we focused on the development of a bispecific antibody approach to target NK cells to EGFR expressing colorectal cancer. The potential future clinical applicability of the outlined NK cell engaging approach could be much broader. On the one hand, the use of the EGFR-CD16 bispecific VHH could be extended to other EGFR expressing tumor types. On the other hand, by fusing the CD16-specific VHH to VHHs against other tumor targets of interest, tumors negative for EGFR could also be targeted. For example, carcinoembryonic antigen (CEA) could represent an interesting tumor target due to its high expression by multiple epithelial tumors (e.g., colon, gastric, pancreatic, breast, and lung cancer) [68]. The human epidermal growth factor receptor 2 (HER2) constitutes another target of interest. It can be expressed by several tumor types, including breast and gastroesophageal cancers [69], but also by a small proportion of KRAS-BRAF-wild type CRC, where it was identified as a mechanism of resistance for EGFR antibody therapies [70]. Bispecific VHHs targeting CD16 and either CEA [71] or HER2 [65] are currently in pre-clinical development. Of interest is also the development of a bispecific antibody targeting both CD16 and CD133, a stem cell marker expressed by various tumor types, including CRC [72]. In this study, we mostly focused on the direct effect of C-7 on NK cell activation and cytotoxicity. However, proinflammatory cytokines produced by activated NK cells could also beneficially impact the antitumor properties of other immune cells in the tumor microenvironment, including CD8^+^ cytotoxic T cells.

In conclusion, the CD16-EGFR bispecific VHH reported here can trigger efficient lysis of EGFR expressing tumor cell lines and patient metastatic colorectal cancer cells and could represent a valid therapeutic strategy either alone or in combination with other NK cell-based therapeutic approaches, such as the anti-NKG2A monoclonal antibody monalizumab [73], or the adoptive transfer of expanded autologous or allogeneic NK cells.

## 5. Conclusions

In this study, we describe the generation and the functional characterization of a bispecific VHH targeting CD16 and EGFR. This bispecific VHH was shown to trigger activation of CD16^+^ NK cells, resulting in enhanced cytotoxicity of EGFR^+^ tumor cell lines and EGFR^+^ patient CRC specimens in both an autologous and allogeneic setting. Based on these results, this novel CD16-EGFR bispecific engager might represent a useful tool either alone or in combination with other NK cell-based therapies.

## Figures and Tables

**Table 1:** Apparent K_d_ of C-7 and 7-C towards CD16 and EGFR.

Cell Type	C-7 Apparent K_d_ (95% CI)	7-C Apparent K_d_ (95% CI)
CD56^+^CD3^−^	0.84 nM (0.32;2.00)	3.00 nM (1.10;7.48)
NK92 CD16^+^	6.80 nM (4.98;9.19)	77.60 nM (48.14; 121.70)
A431	196.1 nM (86.38;460.2)	29.56 nM (11.92;79.69)

## Data Availability

The data presented in this study are available on request from the corresponding author. The data are not publicly available due to privacy-related issues.

## Supplementary Materials

The following are available online at https://www.mdpi.com/article/10.3390/cancers13215446/s1, Figure S1: Gating strategy used to identify binding of the 7-C and C-7 bispecific VHH and a CD16 specific monoclonal antibody to CD56^+^CD3^−^ NK cells in PBMC. Figure S2: Expression of EGFR and NK cell activating and inhibitory ligands on A431, HCT116, and Colo829. Figure S3: Degranulation of CD3^+^ T cells after a 4-h co-culture of CRC patient derived monocyte-depleted PBMC and A431 tumor cells. Figure S4: Representative example of gating strategy used to assess tumor cell cytotoxicity induced during a co-culture of dissociated patient CRC peritoneal metastatic cells and autologous PBMC. Figure S5: Distribution of CD16^+^ immune cells in peritoneal CRC tumor suspensions. Figure S6: Efficiency of CD14 depletion through magnetic bead-activated cell sorting of PBMC derived from patients with CRC. Table S1: Characteristics of all patients and healthy donors included in the study. Table S2: Catalogue numbers and clones of used antibodies.
