# Supplementary material for: Enhancement of NK Cell Antitumor Effector Functions Using a Bispecific Single Domain Antibody Targeting CD16 and the Epidermal Growth Factor Receptor
Source: Cancers (Basel). 2021 Oct 29;13(21):5446. doi: 10.3390/cancers13215446 (PMC8582566; doi:10.3390/cancers13215446)
Supplement: Supplementary file 1 [file cancers-13-05446-s001.zip › cancers-1433410-supplementary.pdf]

Supplementary

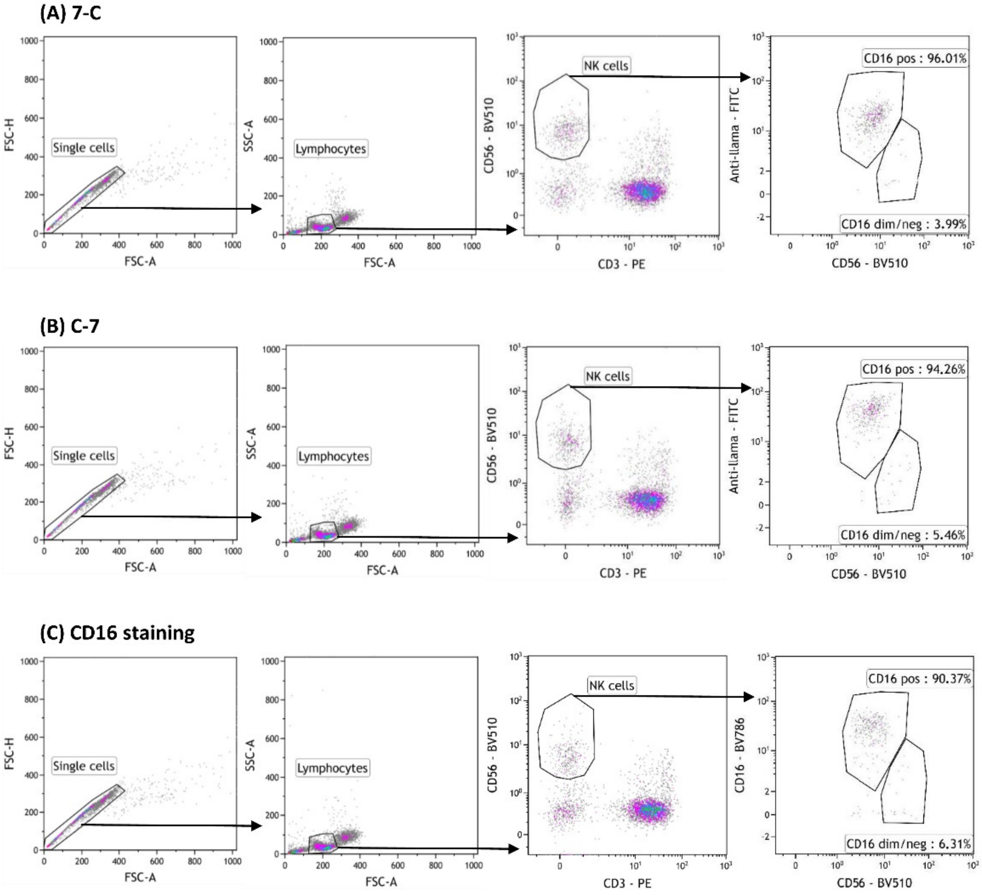

Figure S1: Gating strategy used to identify binding of the 7-C (A) and C-7 (B) bispecific VHH and a CD16 specific monoclonal antibody (C) to CD56^{+}CD3^{-} NK cells in PBMC.

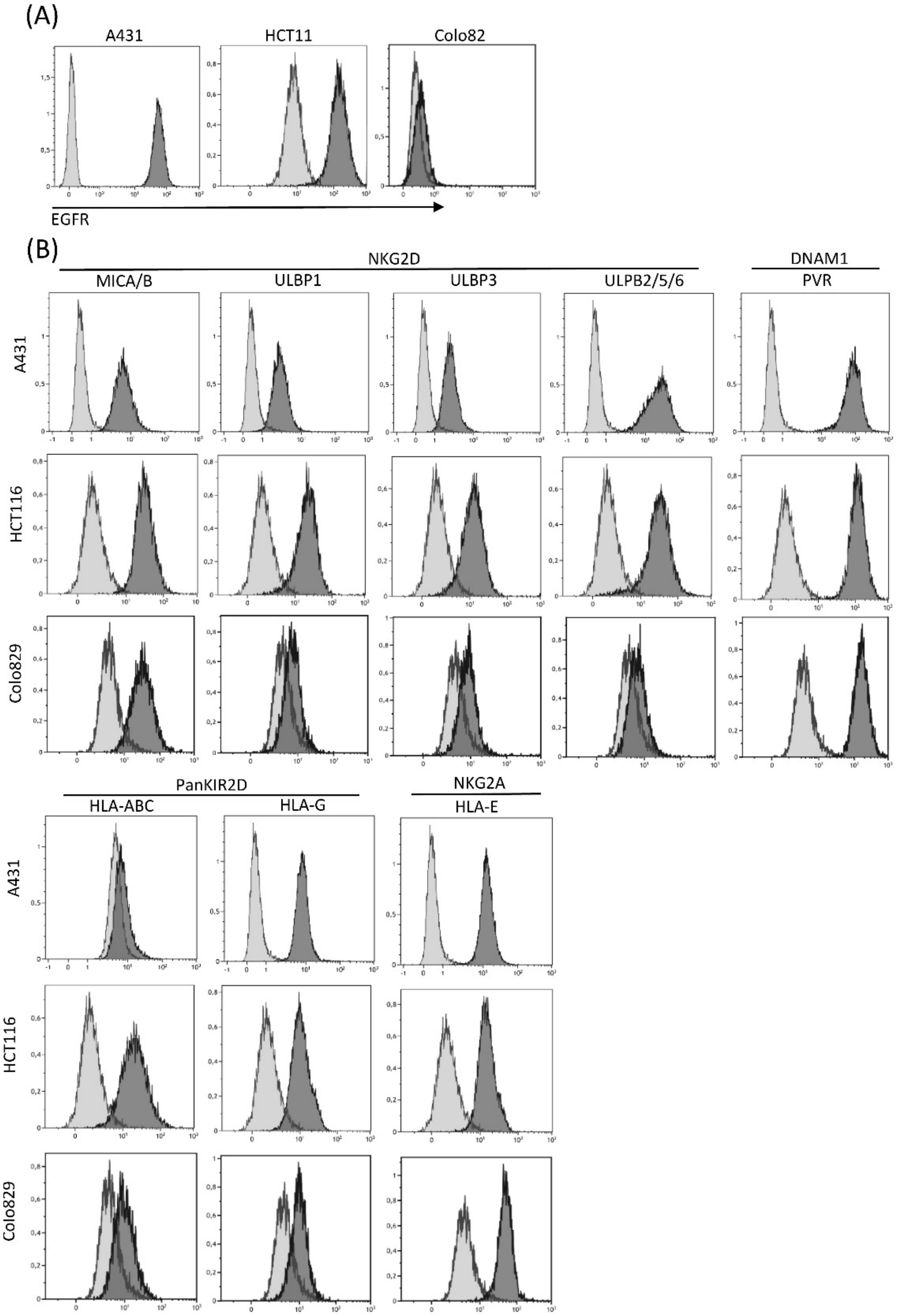

Figure S2. (A) Expression of EGFR and **(B)** NK cell activating and inhibitory ligands on A431, HCT116, and Colo829. Light grey histograms: unstained control; dark grey histograms: marker expression.

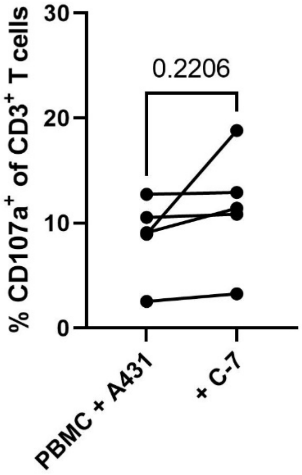

Figure S3. Degranulation of CD3⁺ T cells after a 4-hour co-culture of CRC patient derived monocyte-depleted PBMC and A431 tumor cells ± 100 nM C-7. E:T ratio 4:1. N=5. The p-value is determined by two-tailed paired t-test.

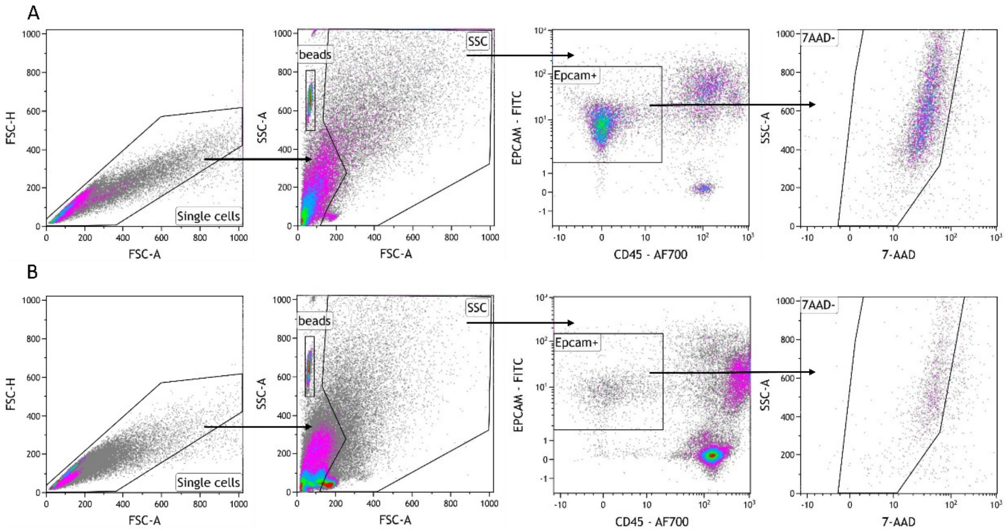

Figure S4. Representative example of gating strategy used to assess tumor cell cytotoxicity induced during a co-culture of dissociated patient CRC peritoneal metastatic cells and autologous PBMC. A) Tumor suspension alone B) Co-culture of tumor suspension with autologous PBMC and C-7.

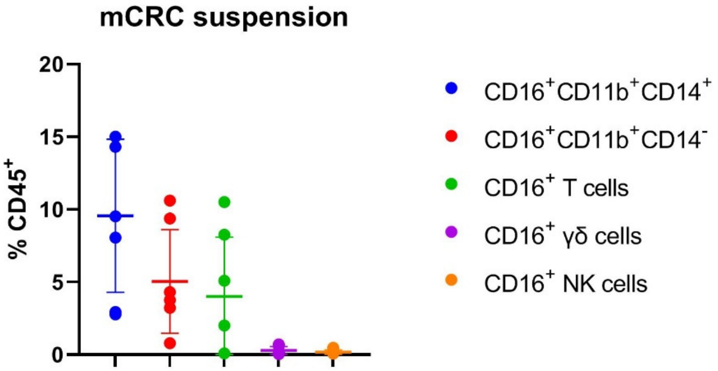

Figure S5. Distribution of $CD16^+$ immune cells in peritoneal CRC tumor suspensions. Expressed as percentages of $CD45^+$ cells. N=7.

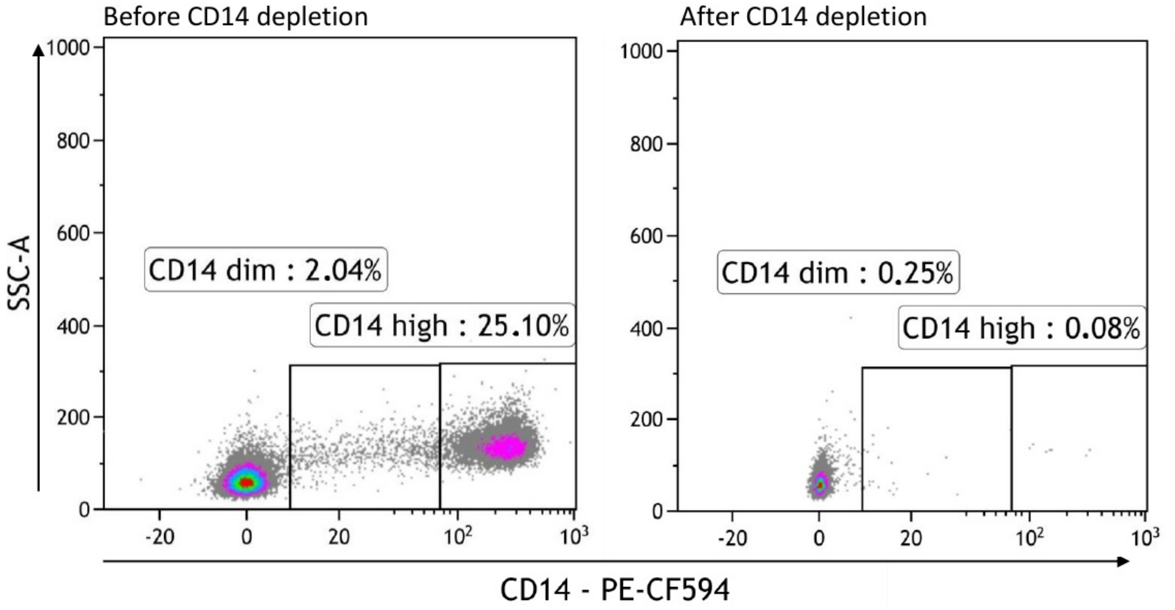

Figure S6. Efficiency of CD14 depletion through magnetic bead-activated cell sorting of PBMC derived from patients with CRC. The $CD14^{dim}$ subset comprises ($CD14^{dim}CD16^{high}$) non-classical monocytes while the $CD14^{high}$ subset includes ($CD14^{high}CD16^{dim}$) intermediate and ($CD14^{high}CD16^{neg}$) classical monocytes[42].

Table S1. Characteristics of all patients and healthy donors included in the study.

	**Patient characteristics (N=21)**	**Healthy donors (N=18)**
	N (%)	N(%)
Gender (man)	9 (42.9)	10 (55.5)
Age*	66.0 (36-80)	33.2 (19-62)
Cancer type		
Colorectal Cancer	21 (100.0)	
Stage		
II	3 (14.3)	

III	2 (9.5)
IV	16 (76.2)
Chemotherapy	
Pretreated	9 (42.9)
Mutational status	
No mutation	1 (4.8)
KRAS mutation	1 (4.8)
BRAF mutation	4 (19.0)
Unknown	15 (71.4)
Microsatellite instability	
MSS or MMR proficient	6 (28.5)
MSI	3 (13.4)
Unknown	12 (57.1)

* Mean (range).

Table S2. Catalogue numbers and clones of used antibodies.

Antibodies	Brand	Catalog number	Clone
CD56 APC-Vio770	Miltenyi	130-114-548	REA196
CD56 CD510	BD Horizon	563041	NCAM16.2
CD3 BV711	BD Horizon	563725	UCHT1
CD3 PE	BD Biosciences	345765	SK7
CD16 BV786	BD Horizon	563690	3G8
Anti-llama FITC	Bioke	BET A160-100F	Policlonal
CD107a PE	ThermoFisher	12-1079-42	eBioH4A3
CD45 AF700	Biolegend	304024	HI30
Epcam FITC	Biolegend	324204	9C4
HLA-E PE	eBioscience	12-9953-41	3D12HLA-E
HLA-G PE	Biolegend	335905	87G
PVR PE	Biolegend	337507	SK11.4
MICA/B PE	Biolegend	320906	6D4
ULBP 2/5/6 PE	R&D systems	FAB1298P	165903
ULBP1 PE	R&D systems	FAB1380P	170818
ULBP3 PE	R&D systems	FAB1517P	166510
HLA-ABC PE	ThermoFisher	12-9983-41	W6/32
EGFR BV421	Biolegend	352911	AY13
CD14 PE-CF594	BD Biosciences	562335	MφP9
γδ TCR BV421	BD Pharmingen	562560	B1
CD11b APC	BD Biosciences	333143	D12
